# Butyrate Prevents the Pathogenic Anemia‐Inflammation Circuit by Facilitating Macrophage Iron Export

**DOI:** 10.1002/advs.202306571

**Published:** 2024-01-18

**Authors:** Peng Xiao, Xuechun Cai, Zhou Zhang, Ke Guo, Yuehai Ke, Ziwei Hu, Zhangfa Song, Yuening Zhao, Lingya Yao, Manlu Shen, Jingyun Li, Youling Huang, Lingna Ye, Lingjie Huang, Yu Zhang, Rongbei Liu, Mengque Xu, Xutao Xu, Yuan Zhao, Qian Cao

**Affiliations:** ^1^ Department of Gastroenterology, Sir Run Run Shaw Hospital Zhejiang University School of Medicine Hangzhou Zhejiang 310016 China; ^2^ Institute of Immunology Zhejiang University School of Medicine Hangzhou Zhejiang 310058 China; ^3^ The Key Laboratory for Immunity and Inflammatory Diseases of Zhejiang Province Hangzhou 310058 China; ^4^ Inflammatory Bowel Diseases Center, Sir Run Run Shaw Hospital Zhejiang University School of Medicine Hangzhou Zhejiang 310016 China; ^5^ Department of Pathology and Pathophysiology Zhejiang University School of Medicine Hangzhou Zhejiang 310058 China; ^6^ Department of Colorectal Surgery, Sir Run Run Shaw Hospital Zhejiang University School of Medicine Hangzhou Zhejiang 310016 China

**Keywords:** anemia, butyrate, ferroportin, inflammatory bowel disease, macrophages

## Abstract

Most patients with inflammatory bowel disease (IBD) develop anemia, which is attributed to the dysregulation of iron metabolism. Reciprocally, impaired iron homeostasis also aggravates inflammation. How this iron‐mediated, pathogenic anemia‐inflammation crosstalk is regulated in the gut remains elusive. Herein, it is for the first time revealed that anemic IBD patients exhibit impaired production of short‐chain fatty acids (SCFAs), particularly butyrate. Butyrate supplementation restores iron metabolism in multiple anemia models. Mechanistically, butyrate upregulates ferroportin (FPN) expression in macrophages by reducing the enrichment of histone deacetylase (HDAC) at the *Slc40a1* promoter, thereby facilitating iron export. By preventing iron sequestration, butyrate not only mitigates colitis‐induced anemia but also reduces TNF‐α production in macrophages. Consistently, macrophage‐conditional FPN knockout mice exhibit more severe anemia and inflammation. Finally, it is revealed that macrophage iron overload impairs the therapeutic effectiveness of anti‐TNF‐α antibodies in colitis, which can be reversed by butyrate supplementation. Hence, this study uncovers the pivotal role of butyrate in preventing the pathogenic circuit between anemia and inflammation.

## Graphical abstract

Anemia is the most common extraintestinal complication in patients with inflammatory bowel disease (IBD). As a short‐chain fatty acid (SCFA), butyrate ameliorates inflammation‐associated anemia by facilitating ferroportin (FPN)‐mediated iron export in macrophages. By this mechanism, butyrate also reduces the production of inflammatory cytokine TNF‐α in macrophages. This study uncovers a new role of butyrate as an inhibitor of the pathological “anemia‐inflammation” cycle.

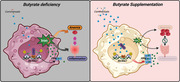

## 1 Introduction

As the most common extraintestinal complication in inflammatory bowel disease (IBD), anemia occurs in a majority of IBD patients. The predominant form of anemia in IBD is iron deficiency anemia (IDA), characterized by low iron and hemoglobin concentrations in the blood and reduced numbers of red blood cells. Typical symptoms of IDA include fatigue, headaches, heart palpitations, and shortness of breath, which substantially reduce the quality of life in IBD patients.^[^ ^1^ ^]^ The etiology of IBD‐associated IDA is multifaceted, such as bleeding, defective iron absorption, or inflammation‐induced abnormal iron metabolism.^[^ ^1b^ ^]^ Nevertheless, how iron homeostasis is regulated in the gut microenvironment remains largely unknown to date, and thus greatly hindered the development of anti‐anemia therapeutic strategies.

Over the past decade, growing evidence has revealed that the dysregulation of host immune cells, particularly macrophages, is a crucial culprit for IDA.^[^ ^2^ ^]^ Reciprocally, abnormal iron metabolism leads to inflammatory activation of immune cells,^[^ ^3^ ^]^ which further aggravates IDA. How to prevent this pathogenic crosstalk between anemia and inflammation is pivotal for relieving the symptoms of IBD patients.

The presence of microbiomes and their metabolites are the distinctive environmental features in the gut. As the most abundant microbial fermentation products, short‐chain fatty acids (SCFAs) are essential for maintaining the epithelium as well as immune homeostasis in the intestine.^[^ ^4^ ^]^ However, whether SCFAs are involved in the regulation of intestinal iron metabolism remains elusive. In this report, we identified butyrate, a four‐carbon SCFA, plays a crucial role in modulating iron homeostasis in the context of colitis through promoting ferroportin (FPN)‐dependent macrophage iron export. This effect not only ameliorated colitis‐associated IDA but also prevented the inflammatory activation of intestinal macrophages. In addition, we uncovered a novel role of iron in limiting the therapeutic efficacy of anti‐TNF‐α antibodies in IBD patients.

## 2 Results

### 2.1 Anemic IBD Patients Exhibit Impaired Butyrate Production

**Figure 1:**
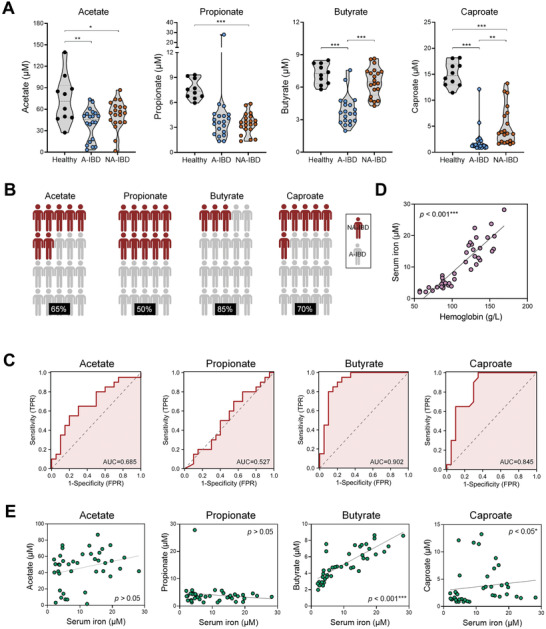
Insufficient SCFA production is correlated with inflammation‐associated anemia in IBD patients. A) The levels of indicated SCFAs in the serum from A‐IBD patients (*n* = 20), NA‐IBD patients (*n* = 20), and healthy controls (*n* = 10) were measured. ^*^ *p* < 0.05; ^**^ *p* < 0.01; ^***^ *p* < 0.001, unpaired, two‐tailed Student's *t*‐test. B) The proportions of A‐IBD and NA‐IBD patients were analyzed in SCFAlow patients (*n* = 20). C) ROC curve analysis indicated the predictive roles of serum SCFAs in distinguishing between A‐IBD and NA‐IBD patients. D) The correlation between hemoglobin and serum iron was analyzed using Spearman's correlation test. D) The correlations between serum iron and SCFA levels in IBD patients were analyzed using Spearman's correlation test, ^*^ *p* < 0.05; ^***^ *p* < 0.001.

First, we investigated the potential association between SCFA production and anemia parameters in IBD. To this end, 40 IBD patients were divided into an anemia group (A‐IBD, including ten ulcerative colitis patients and ten Crohn's disease patients) and a non‐anemia group (NA‐IBD, also including ten ulcerative colitis patients and ten Crohn's disease patients) base on their hemoglobin levels. To achieve a better separation, A‐IBD individuals and NA‐IBD individuals were defined as hemoglobin levels <105 g L^−1^ and hemoglobin levels >115 g L^−1^, respectively. Patients’ characteristics are listed in Table S1 (Supporting Information). Compared with healthy controls, IBD patients exhibited a general reduction of serum SCFA levels, including acetate, propionate, butyrate, and caproate. The levels of other SCFAs, including isobutyrate, pentanoate, and isopentanoate, were below the detection threshold in a portion of subjects, particularly in IBD patients (data not shown). Notably, the serum levels of butyrate and caproate were significantly higher in NA‐IBD patients than those in A‐IBD patients (**Figure** **1**A). The higher butyrate concentration in NA‐IBD patients than in A‐IBD patients was observed in both female and male cohorts (Figure S1, Supporting Information). When we divided patients based on their SCFA concentrations, 85% of patients with low butyrate production were anemic, while this percentage for acetate, propionate, and caproate was 65%, 50%, and 70%, respectively (Figure 1B). In addition, butyrate, and to a lesser extent, acetate, and caproate, exhibited high accuracy in distinguishing between the A‐IBD and NA‐IBD patients. The areas under the receiver operating characteristic (ROC) curves were 0.902 (butyrate), 0.685 (acetate), and 0.845 (caproate) respectively (Figure 1C).

As anticipated, there is a strong positive correlation between hemoglobin and serum iron levels, indicating that abnormal iron metabolism is responsible for IBD‐associated anemia (Figure 1D). Importantly, among the SCFAs, serum butyrate levels exhibited the most robust positive correlation with serum iron levels (Figure 1E). These findings indicate that SCFAs, particularly butyrate, may play a role in maintaining host iron metabolism during intestinal inflammation.

### 2.2 Butyrate Ameliorates Anemia by Normalizing Iron Homeostasis

**Figure 2:**
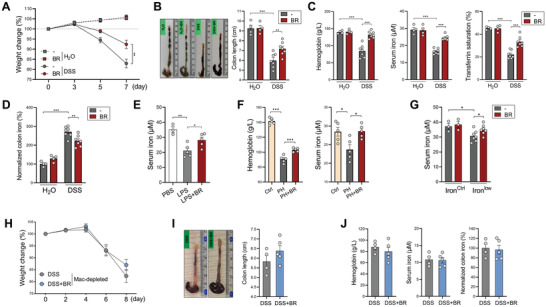
Butyrate prevents anemia in colitis by maintaining iron homeostasis. A,B) Mice were fed 2.5% DSS with or without 150 mm butyrate in drinking water. Body weight change (A) and colon length (B) were measured (*n* = 4 for H_2_O groups, *n* = 7 for DSS groups). C) The levels of hemoglobin, serum iron, and transferrin saturation were evaluated. D) Iron levels in colon tissues were evaluated. E) Mice were pretreated with 150 mm sodium butyrate in drinking water for two days, followed by intraperitoneal (i.p.) injection of 5 mg k^−1^g LPS for 6 h. The levels of serum iron were measured (*n* = 3 to 5 per group). F) Control or butyrate‐administered mice were intraperitoneally injected with 40 mg k^−1^g PHZ, the levels of hemoglobin and serum iron were evaluated 2 days after injection (*n* = 5 per group). G) Mice were fed on a low iron diet (8 ppm, *n* = 7 per group) or control diet (45 ppm, *n* = 3 per group) for four weeks, with or without 100 mm butyrate in drinking water. The levels of serum iron were measured. H–J) Mice were intraperitoneally injected with clodronate liposomes to deplete macrophages, followed by 2.5% DSS challenge with or without butyrate administration (*n* = 4 to 5 per group). Body weight change (H) and colon length (I) were measured. J) The levels of hemoglobin, serum iron, and colonic iron were evaluated. ^*^ *p* < 0.05; ^**^ *p* < 0.01; ^***^ *p* < 0.001, unpaired, two‐tailed Student's *t*‐test.

To further clarify how butyrate impacts colitis‐associated anemia, we subjected mice to a DSS‐induced colitis model with butyrate given in drinking water, a well‐adopted method for butyrate supplementation in animal models.^[^ ^5^ ^]^ As shown in **Figure** **2**A,B, butyrate significantly alleviated colitis as evidenced by improved weight loss and colon shortening. Compared to healthy mice, colitic mice exhibited reduced levels of hemoglobin, serum iron, and transferrin saturation. Notably, these symptoms of anemia were significantly alleviated by butyrate (Figure 2C). In contrast to the reduced serum iron levels, the contents of iron in the colon were significantly increased in colitic mice, and this colitis‐induced iron overload was significantly reversed by butyrate (Figure 2D). Apart from intestinal inflammation, butyrate also prevented the decrease in serum iron in an LPS‐induced sepsis model (Figure 2E).

To investigate whether the anti‐anemic effect of butyrate is only secondary to reduced inflammation, we established an inflammation‐independent anemia model by injecting mice with phenylhydrazine (PHZ), which leads to red cell damage and the consequent hemoglobin lease. As shown in Figure 2F, butyrate significantly mitigated the reduction of hemoglobin and serum iron in PHZ‐treated mice. We further adopted an additional anemia model by feeding mice a low‐iron diet. Again, butyrate administration effectively improved the serum iron levels in mice given a low‐iron diet (Figure 2G), indicating that the anti‐anemic function of butyrate is achieved by directly maintaining iron homeostasis.

Similar to immunocompetent mice, treatment of athymic nude mice with butyrate significantly improved hemoglobin and serum iron levels, and prevented colonic iron overload in colitis mice, suggesting that adaptive immune cells are dispensable for the anti‐anemic capacity of butyrate (Figure S2, Supporting Information). Macrophages play a predominant role in controlling the host's iron homeostasis,^[^ ^2^, ^6^ ^]^ we found that depletion of macrophages with clodronate liposomes largely abrogated the anti‐colitic function of butyrate (Figure 2H). Importantly, in macrophage‐depleted mice, butyrate failed to alter hemoglobin concentrations, and the contents of serum iron and colonic iron (Figure 2J). Thus, butyrate maintains host iron homeostasis by acting on macrophages.

### 2.3 Butyrate Facilitates FPN Expression and FPN‐Dependent Iron Export in Macrophages

**Figure 3:**
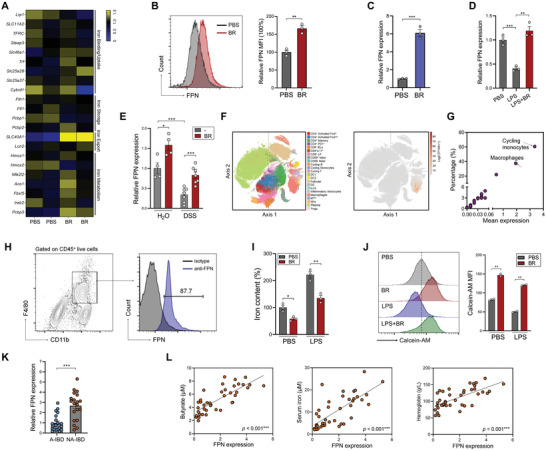
Butyrate prevents iron overload in macrophages by promoting FPN expression. A) Peritoneal macrophages (PMs) were treated with 2 mm butyrate for 12 h, RNA sequencing was performed. The levels of iron metabolism‐related genes were analyzed. B) PMs were treated with 1 mm butyrate for 12 h, the protein levels of FPN were evaluated by flow cytometry. C) Human PBMC‐derived macrophages were treated with 2 mm butyrate for 12 h, FPN expression was evaluated by QPCR. D) PMs were pretreated with 1 mm butyrate followed by 1 µg mL^−1^ LPS stimulation for 12 h, FPN expression was evaluated by QPCR. E) Mice were fed 2.5% DSS with or without 150 mm butyrate in drinking water, the expression of FPN in colonic macrophages was evaluated by QPCR. (*n* = 4 to 7 per group). F) FPN expression in human colon immune cells was explored using Single Cell Portal (https://singlecell.broadinstitute.org/single_cell, accession number SCP259). G) The quantification of monocyte and macrophage FPN expression in (F). H) Mice were fed 2.5% DSS for 8 days, the protein levels of FPN on CD45^+^CD11b^+^F4/80^+^ colonic macrophages were evaluated by flow cytometry. I,J) PMs were pretreated with 1 mm butyrate for 4 h, followed by 1 µ g mL^−1^ LPS stimulation overnight. The intracellular iron contents were measured (I), the fluorescence intensity of calcein‐AM was evaluated by flow cytometry (J). K) The expression levels of FPN in the colonic mucosa from A‐IBD and NA‐IBD patients were evaluated by QPCR (*n* = 20 per group). ^*^ *p* < 0.05; ^**^ *p* < 0.01; ^***^ *p* < 0.001, unpaired, two‐tailed Student's *t*‐test. L) The correlations between mucosal FPN expression and serum butyrate, serum iron, and hemoglobin concentrations in IBD patients (*n* = 40) were analyzed by Spearman's correlation test, ^***^ *p* < 0.001.

To investigate how butyrate modulates iron metabolism in macrophages, we subjected control and butyrate‐treated peritoneal macrophages to RNA sequencing. Among the genes related to iron metabolism, we observed that the expression of ferroportin (FPN, encoded by *Slc40a1* gene), the only known iron exporter, was significantly increased by butyrate (**Figure** **3**A). Butyrate dose‐dependently enhanced the mRNA expression of FPN in macrophages (Figure S3, Supporting Information), and the butyrate‐induced upregulation of FPN was further confirmed by flow cytometry (Figure 3B). Similar to peritoneal macrophages, butyrate significantly induced FPN expression in macrophages of multiple tissue origins, including colonic macrophages, splenic macrophages, and hepatic macrophages (Figure S4, Supporting Information). The FPN‐inducing capacity of butyrate was also verified in human PBMC‐derived macrophages (Figure 3C). In addition, butyrate restored the LPS‐induced suppression of FPN (Figure 3D). Compared to healthy mice, colitic mice exhibited significantly reduced levels of FPN in colonic macrophages, which were rescued by butyrate supplementation (Figure 3E). We further explored a single‐cell RNA sequencing dataset and found that among *lamina propria* immune cells in the human colon, monocytes and macrophages express the highest levels of FPN (Figure 3F,G). Through performing flow cytometry, we found that FPN was expressed in more than 85% of CD11b^+^F4/80^+^ colonic macrophages (Figure 3H). The protein levels of FPN on the surface of colonic macrophages were significantly increased by butyrate supplementation in colitic mice (Figure S5, Supporting Information).

Consistent with the elevated FPN expression, butyrate treatment significantly reduced the intracellular iron contents in resting and LPS‐stimulated macrophages (Figure 3I). The iron‐exporting effect of butyrate was further confirmed via calcein‐AM staining (higher calcein‐AM intensity means lower intracellular iron content) (Figure 3J). Compared to butyrate, other SCFAs had only a weak or marginal effect on intracellular iron contents in macrophages (Figure S6, Supporting Information).

In line with their higher serum iron levels, NA‐IBD patients exhibited significantly higher FPN expression in the colonic mucosa than A‐IBD patients (Figure 3K). More importantly, the expression of mucosal FPN showed significant positive correlations with butyrate production, serum iron levels, and hemoglobin in IBD patients (Figure 3L). Taken together, butyrate‐mediated FPN induction is crucial for preventing iron overload in macrophages.

### 2.4 Macrophage‐Conditional FPN Depletion Aggravates Colitis‐Associated Iron Disequilibrium

**Figure 4:**
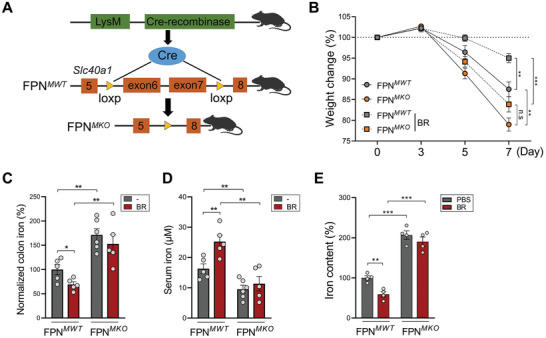
FPN deficiency in macrophages aggravates colitis‐associated iron disequilibrium. A) Construction strategy of the FPN*^MKO^* mice. B) FPN*^MWT^* and FPN*^MKO^* mice were fed 2.5% DSS with or without 150 mm butyrate in drinking water (*n* = 5 to 6 per group). Body weight changes were measured. C) The levels of serum iron were evaluated. D) The levels of colonic iron were evaluated. E) PMs from FPN*^MWT^* and FPN*^MKO^* mice were pretreated with 1 mm butyrate for 4 h, followed by 1 µ g mL^−1^ LPS stimulation overnight. The intracellular iron contents were measured. ^*^ *p* < 0.05; ^**^ *p* < 0.01; ^***^ *p* < 0.001, unpaired, two‐tailed Student's *t*‐test.

To further elucidate the impact of macrophage‐expressed FPN in colitis, we generated macrophage‐conditional FPN knockout mice (FPN*^MKO^*) by crossing LysM‐Cre mice with *FPN* ^flox/flox^ mice (**Figure** **4**A). As shown in Figure 4B, FPN*^MKO^* mice developed significantly more severe colitis than FPN*^MWT^* mice. Although butyrate improved colitis in FPN*^MWT^* mice, this effect was partially compromised in FPN*^MKO^* mice (Figure 4B), suggesting that inducing macrophage FPN expression serves as a crucial protective mechanism of butyrate.

The colonic iron contents were significantly higher in FPN*^MKO^* mice compared to FPN*^MWT^* mice after the DSS challenge. Importantly, unlike in FPN*^MWT^* mice, butyrate failed to normalize the colonic iron overload in FPN*^MKO^* mice (Figure 4C). As expected, we observed significantly reduced serum iron levels in FPN*^MKO^* mice, which could not be reversed by butyrate administration (Figure 4D).

In line with the animal experiments, macrophages from FPN*^MKO^* mice had significantly higher iron contents after LPS stimulation. Again, butyrate only improved the intracellular iron sequestration in FPN*^MWT^* macrophages, but not in FPN*^MKO^* macrophages (Figure 4E; Figure S7, Supporting Information). These findings demonstrate that butyrate maintains iron homeostasis in colitis by modulating macrophage FPN expression.

### 2.5 Butyrate Blocks the Pathogenic Circuit Between Anemia and Inflammation by Increasing Iron Export

**Figure 5:**
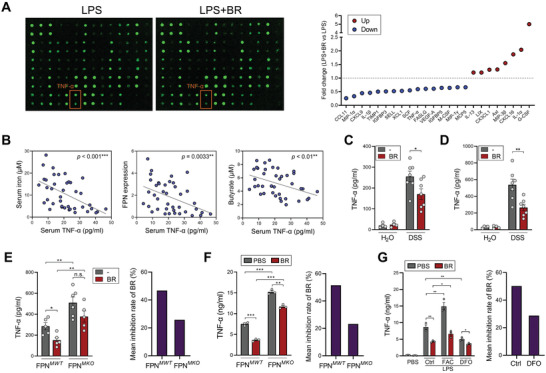
Butyrate inhibits TNF‐α production in macrophages by facilitating iron export. A) PMs were pre‐treated with butyrate for 6 h, followed by 1 µ g mL^−1^ LPS stimulation for 12 h, the culture supernatants were harvested and were subjected to a cytokine array. Orange boxes indicate TNF‐α (left). Fold changes (LPS + BR vs LPS) were quantified (right). B) The correlations between serum TNF‐α and serum iron, mucosal FPN expression, or serum butyrate concentrations in IBD patients (*n* = 40) were analyzed using Spearman's correlation test. ^**^ *p* < 0.01; ^***^ *p* < 0.001. C,D) The protein levels of TNF‐α in colon homogenates (C) and culture supernatants of colonic macrophages (D) from DSS‐fed mice were evaluated by ELISA (*n* = 7 per group). E) The protein levels of TNF‐α in colon homogenates from FPN*^MWT^* and FPN*^MKO^* mice were evaluated by ELISA (*n* = 5 to 6 per group, left). The mean inhibition rates of butyrate in FPN*^MWT^* and FPN*^MKO^* mice were calculated (right). F) PMs from FPN*^MWT^* and FPN*^MKO^* mice were pretreated with PBS or 1 mm butyrate for 6 h, followed by 1 µg mL^−1^ LPS stimulation for 6 h. The levels of TNF‐α in culture supernatants were evaluated by ELISA (left). The mean inhibition rates of butyrate in FPN*^MWT^* and FPN*^MKO^* macrophages were calculated (right). G) PMs were pretreated with PBS or 1 mm butyrate for 6 h, followed by 1 µg mL^−1^ LPS stimulation for 6 h in the presence of FAC (100 µm) or DFO (40 µm). The levels of TNF‐α in culture supernatants were evaluated by ELISA (left). The mean inhibition rates of butyrate in control or DFO‐treated groups were calculated (right). ^*^ *p* < 0.05; ^**^ *p* < 0.01; ^***^ *p* < 0.001, unpaired, two‐tailed Student's *t*‐test.

Iron overload is well‐known to increase the production of inflammatory cytokines in macrophages^[^ ^2^, ^7^ ^]^ and worsen colitis.^[^ ^8^ ^]^ Contrarily, iron chelation reduced the severity of intestinal inflammation.^[^ ^9^ ^]^ Through performing a cytokine array, butyrate was found to suppress the production of several pro‐inflammatory cytokines including IL‐1β, TNF‐α, CXCL9, and CCL11, whereas upregulated the levels of anti‐inflammatory cytokines such as IL‐13 and CX3CL1 (**Figure** **5**A). We selected TNF‐α as the primary indicator in our following work since it is the most clinically relevant colitogenic cytokine.^[^ ^10^ ^]^ Additionally, TNF‐α monoclonal antibody is one of the most commonly used drugs in the clinical treatment of colitis.^[^ ^11^ ^]^

In IBD patients, the serum levels of TNF‐α exhibited significantly negative correlations with serum iron, mucosal FPN expression, and serum butyrate concentrations (Figure 5B), indicating the close association between iron dysregulation and inflammation. Consistent with its FPN‐inducing role in macrophages, butyrate administration significantly reduced TNF‐α production in colon tissues and colonic macrophages from colitic mice (Figure 5C,D).

We further examined whether macrophage iron export could affect TNF‐α production in colitis. In line with the impaired iron export in FPN*^MKO^* mice, the colonic TNF‐α levels were significantly elevated in FPN*^MKO^* mice than in FPN*^MWT^* mice. On the other hand, although butyrate robustly suppressed TNF‐α production in FPN*^MWT^* mice, it only mildly reduced colonic TNF‐α levels in FPN*^MKO^* mice. Quantitative analysis demonstrated that the TNF‐α suppressive capacity of butyrate was compromised in FPN*^MKO^* mice (Figure 5E), indicating that macrophage FPN expression is a key mediator for the anti‐inflammatory effect of butyrate. In line with the in vivo data, macrophages from FPN*^MKO^* mice produced significantly higher amounts of TNF‐α after LPS stimulation. Notably, the capacity of butyrate to reduce TNF‐α production was compromised in FPN‐depleted macrophages (Figure 5F).

On the other hand, ferric ammonium citrate (FAC) treatment significantly elevated TNF‐α production in macrophages, whereas butyrate significantly reduced TNF‐α in both LPS and LPS+FAC‐treated macrophages. In contrast to iron overload, iron chelation by deferoxamine (DFO) inhibited TNF‐α production as previously reported.^[^ ^2^, ^7^, ^12^ ^]^ The TNF‐α suppressive capacity of butyrate was compromised in the presence of DFO, suggesting that they are functionally redundant to some extent in preventing cellular iron overload (Figure 5G). Taken together, facilitating iron export is crucial for the anti‐inflammatory function of butyrate.

### 2.6 Preventing Iron Overload Sensitizes the Therapeutic Efficacy of Anti‐TNF‐α Antibody

**Figure 6:**
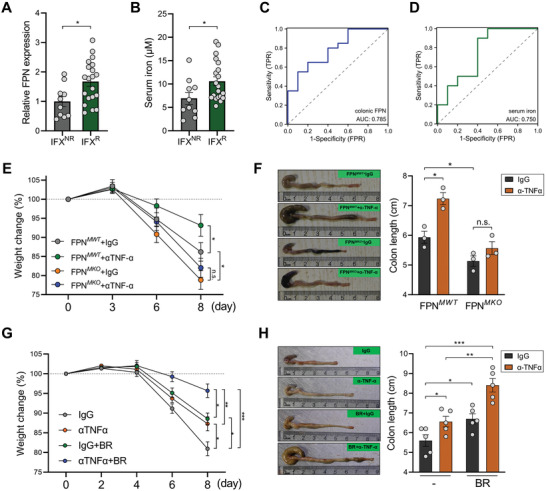
FPN‐mediated macrophage iron export is required for the optimal response to αTNF‐α therapy. A) The serum iron levels in IFX^NR^ (*n* = 10) and IFX^R^ IBD patients (*n* = 20) were evaluated. B) The expression levels of mucosal FPN in IFX^NR^ and IFX^R^ IBD patients were evaluated by QPCR. C,D) ROC curve analysis indicated the predictive roles of mucosal FPN expression (C) or serum iron (D) in distinguishing between IFX^NR^ and IFX^R^ IBD patients. E,F) FPN*^MWT^* and FPN*^MKO^* mice were fed 2.5% DSS and were treated with IgG or αTNF‐α. Body weight change was evaluated (E), colon length was measured on day 8 (F). G,H) Mice fed with 2.5% DSS were treated with IgG or αTNF‐α, with or without 150 mm butyrate in drinking water. Body weight change was evaluated (G), colon length was measured on day 8 (H). ^*^ *p* < 0.05; ^**^ *p* < 0.01; ^***^ *p* < 0.001, unpaired, two‐tailed Student's *t*‐test.

Our above findings have revealed the impact of butyrate on macrophage iron export in colitis. Intriguingly, several previous reports have found that a high abundance of butyrate‐ or SCFA‐producing bacteria was associated with better responsiveness to anti‐TNF‐α (αTNF‐α) therapy in IBD patients.^[^ ^13^ ^]^ This prompts us to investigate whether macrophage iron metabolism could affect the therapeutic efficacy of TNF‐α blockade. To this end, we collected serums from IBD patients who received infliximab (IFX) therapy before treatment and divided them into primary responders (IFX^R^) and primary non‐responders (IFX^NR^) based on their therapeutic outcome. The results showed that IFX^NR^ patients had significantly lower FPN expression in the colonic mucosa than IFX^R^ patients (**Figure** **6**A). In agreement, the serum iron levels were also significantly lower in IFX^NR^ patients (Figure 6B). The area under the ROC curve for the prediction of IFX responsiveness were 0.785 (colonic FPN expression) and 0.750 (serum iron level) respectively, demonstrating the accuracy of host iron status in distinguishing between IFX^R^ and IFX^NR^ cohorts (Figure 6C,D).

Finally, we tested whether macrophage iron export could impact the response to αTNF‐α therapy. To this end, we treated FPN*^MWT^* and FPN*^MKO^* mice with αTNF‐α. Although TNF‐α neutralization significantly mitigated colitis development in FPN*^MWT^* mice, the therapeutic efficacy was compromised in FPN*^MKO^* mice (Figure 6E,F), suggesting that macrophage iron overload limits the effect of αTNF‐α therapy. To further investigate whether increasing FPN expression could amplify the function of αTNF‐α, we treated colitis mice with αTNF‐α alone or in combination with butyrate. In contrast to macrophage FPN depletion, butyrate administration significantly sensitized colitis mice to αTNF‐α (Figure 6G,H). Hence, the FPN‐mediated iron export in macrophages is required for the optimal therapeutic response to TNF‐α blockade.

### 2.7 Butyrate Upregulates FPN Expression by Disrupting HDAC1‐Specificity Protein 1 (Sp1) Interaction

**Figure 7:**
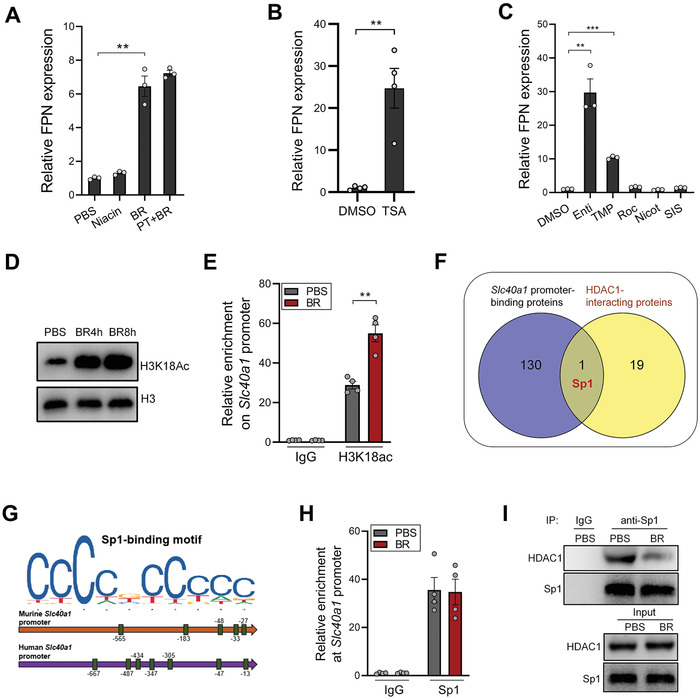
Butyrate increases FPN expression via inhibiting HDAC1‐Sp1 interaction. A) PMs were treated with 2 mm butyrate for 12 h after a 2‐h pertussis toxin (PT) pretreatment, or treated with 1 mm niacin for 10 h, FPN expression was evaluated by QPCR. B) PMs were treated with TSA for 12 h, FPN expression was evaluated by QPCR. C) PMs were treated with HDAC subtype inhibitors (all at 5 µm) for 12 h, FPN expression was evaluated by QPCR. D) PMs were treated with 1 mm butyrate for indicated times, the levels of H3K18Ac were evaluated by immunoblotting. E) PMs were treated with PBS or 1 mm butyrate for 4 h. ChIP assay was performed to assess the levels of H3K18Ac at *Slc40a1* promoter. F) Venn diagram showing proteins that can potentially interact with both human *Slc40a1* promoter and HDAC1. G) Sp1 binding sites within the *Slc40a1* promoter were predicted using the JASPAR database. H) PMs were treated with PBS or 1 mm butyrate for 4 h. ChIP assay was performed to assess the enrichment of Sp1 at *Slc40a1* promoter. I) Peritoneal macrophages were treated with PBS or 1 mm butyrate for 4 h, the interaction between Sp1 and HDAC1 was evaluated by Co‐IP. ^**^ *p* < 0.01; ^***^ *p* < 0.001, unpaired, two‐tailed Student's *t*‐test.

Butyrate is known to regulate gene expression through activating GPCR signaling or inhibiting histone deacetylase (HDAC).^[^ ^14^ ^]^ Blocking GPCR signaling with pertussis toxin did not compromise the capacity of butyrate to upregulate FPN in macrophages. Besides, stimulation with niacin, another GPCR agonist, failed to induce FPN expression (**Figure** **7**A). On the other hand, a pan‐HDAC inhibitor–TSA, recapitulated the FPN‐inducing function of butyrate (Figure 7B). Further results showed that among the five HDAC subtypes, the inhibition of class I HDACs (HDAC1/2/3) by entinostat exhibited the most robust FPN‐inducing capacity, whereas the inhibition of other HDAC classes had a relatively minor effect or no effect (Figure 7C). As expected, butyrate treatment robustly increased histone H3 acetylation in macrophages (Figure 7D). To further explore the effect of butyrate on histone H3 acetylation at *Slc40a1* promoter, a chromatin Immunoprecipitation (ChIP) assay was performed. Indeed, butyrate treatment significantly increased the enrichment of acetyl‐histone H3 at the promoter region of the *Slc40a1* gene (Figure 7E), confirming that butyrate induces FPN expression via its HDAC inhibitory capacity.

We next wondered how butyrate‐mediated HDAC inhibition increases FPN expression. Through performing sequence analysis using the UCSC/JASPAR database, 131 proteins were predicted to bind to the *Slc40a1* promoter. We further identified proteins that interact with HDAC1 using the STRING database. Notably, Sp1 was found to be the only overlapping protein in the two datasets (Figure 7F). In previous work, HDAC inhibitors were reported to disrupt HDAC‐Sp1 interaction at the promoters of specific genes.^[^ ^15^ ^]^ We identified several GC‐rich Sp1‐binding sites within both human and murine *Slc40a1* promoter regions (Figure 7G). The binding of Sp1 to the *Slc40a1* promoter was further validated via a ChIP assay, and Sp1 enrichment at the *Slc40a1* promoter was not affected by butyrate treatment (Figure 7H). On the other hand, through conducting a Co‐Immunoprecipitation (Co‐IP) assay, we found that the interaction between Sp1‐HDAC1 was compromised by butyrate treatment in macrophages (Figure 7I). Therefore, butyrate increased FPN expression by disrupting Sp1‐HDAC1 interaction at the *Slc40a1* promoter.

**Figure 8:**
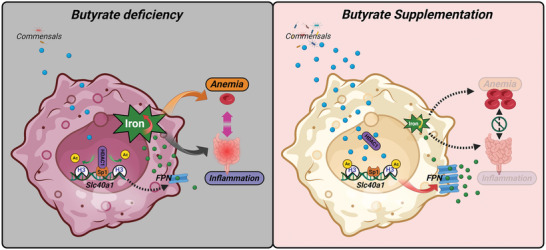
Model illustrating the mechanism that butyrate prevents the vicious cycle between anemia and inflammation in the gut.

In summary, butyrate supplementation rectifies the abnormal iron‐inflammation axis in the gut (**Figure** **8**).

## 3 Discussion

The disruption of iron homeostasis occurs in a variety of inflammatory diseases, resulting in both anemia and immune abnormality. How to impede this pathological “anemia‐inflammation” vicious cycle is of greatest importance for the clinical treatment of inflammation. At present, the treatment approaches for IBD‐associated anemia include iron‐containing oral supplements or intravenous iron preparations. However, the therapeutic efficacy of externally administered iron is unsatisfactory due to its limited bioavailability. In addition, external iron supplementation has a high probability of causing adverse effects, such as abdominal pain, increased disease activity, disrupted gut bacterial diversity, or even anaphylaxis and cardiac arrest.^[^ ^16^ ^]^ Preclinical models have also demonstrated that excessive iron intake leads to worsened colitis,^[^ ^8^ ^]^ which could be protected by iron chelation.^[^ ^9^ ^]^ Interestingly, there is also evidence that iron supplementation inhibited colitis via enhancing intestinal barrier function,^[^ ^17^ ^]^ indicating the complex impacts of iron metabolism on colitis. Apart from iron supplements, recombinant erythropoietin (Epo) is also used for the treatment of IBD‐associated anemia,^[^ ^18^ ^]^ whereas side effects such as the increased risk of thromboembolic events, stroke, or cardiovascular disorders were observed in some patients.

In our work, we revealed the anti‐anemic role of butyrate whose function is achieved by enhancing iron pumping out from macrophages, which also serve as major producers of inflammatory cytokines in colitis. Additionally, butyrate also restored iron metabolism in an LPS‐induced sepsis model. In support of this, butyrate unregulated FPN in macrophages of various tissue origins, including hepatic macrophages and splenic macrophages, which phagocytize senescent and damaged erythrocytes for iron recycling.^[^ ^19^ ^]^ Hence, the anti‐anemic function of butyrate may not be restricted to intestinal inflammation. The data from FPN*^MKO^* mice and macrophage‐depletion experiment demonstrated that macrophages are the primary effector cells responsible for the anti‐anemic function of butyrate, although the contribution of other types of cells cannot be ruled out. It is noteworthy that iron deficiency led to a reduced abundance of SCFA‐producing bacteria, while an iron‐enriched diet upregulated cecal butyrate concentrations.^[^ ^20^ ^]^ Hence, there probably exists a pathogenic circuit where restricted iron availability causes impaired butyrate production, which in turn disrupts host iron recycling.

The mechanistic study revealed that the FPN‐inducing effect is dependent on its HDAC inhibitor activity, especially class I HDACs. In previous work, an HDAC1 inhibitor‐entinostat, was found to decrease serum iron levels in mice by increasing hepcidin expression.^[^ ^21^ ^]^ However, in a clinical trial, patients with end‐stage renal disease given soluble dietary fiber mixture had markedly elevated serum butyrate levels and improved anemia symptoms. In addition, serum butyrate levels exhibited significant positive correlations with serum iron and hemoglobin concentrations.^[^ ^22^ ^]^ This discrepancy might be attributed to several reasons. For example, we and Li et al. administered butyrate or fiber through oral administration. The differential routines may lead to distinct drug absorption and metabolism. Second, although both butyrate and entinostat can act as HDACi, they have distinct chemical properties and metabolic activities. Fortunately, as an endogenous and safe metabolite, butyrate supplementation can be achieved by several means, such as sodium butyrate capsules, butyrate enemas, fecal microbiota transplantation (FMT), or the administration of butyrate‐producing probiotics,^[^ ^5^, ^23^ ^]^ permitting the convenient clinical application.

Although the anti‐inflammatory function of HDAC inhibitors has been previously recognized, this effect is somewhat perplexing because HDAC inhibition typically leads to transcriptional activation. Here, we revealed that iron export at least partially mediated the butyrate‐induced suppression of inflammatory cytokine production in macrophages. Iron overload in macrophages not only aggravated inflammatory responses, but also reduced the efficacy of αTNF‐α therapy. This is supported by the finding that FPN*^MKO^* mice showed reduced responsiveness to TNF‐α blockade. Consistently, patients with lower serum iron levels are less likely to benefit from infliximab treatment. Thus, serum iron levels might be considered a potential marker that predicts the therapeutic outcome of αTNF‐α therapy. We and others have reported that IL‐10 signaling in macrophages not only plays a pivotal anti‐colitic role but is also required for the therapeutic effect of αTNF‐α therapy.^[^ ^24^ ^]^ However, we failed to find an obvious effect of butyrate on macrophage IL‐10 signaling, including the expression of IL‐10 or IL‐10 receptor, IL‐10‐induced STAT3 phosphorylation, and IL‐10‐mediated anti‐inflammatory effect. Therefore, the mechanisms by which iron restrains αTNF‐α function remain elusive and thus await further investigation.

Taken together, our work provides new insights into the biological function of butyrate, which could be harnessed to prevent the vicious cycle between anemia and inflammation.

## 4 Experimental Section

### Mice

Six‐to‐eight‐week‐old, male C57BL/6 wild‐type mice and Balb/c nude mice were purchased from Hangzhou Ziyuan Experimental Animal Technology Co., Ltd. (Hangzhou, China) and housed under specific‐pathogen‐free conditions. *FPN* ^flox/flox^ mice were generously provided by Professor Fudi Wang at Zhejiang University School of Medicine. *FPN* ^flox/flox^ mice were crossed with *LysM‐Cre* mice to generate *LyM‐Cre/FPN* ^flox/flox^ mice (FPN*^MKO^* mice). Age‐ and sex‐matched *FPN* ^flox/flox^ mice (FPN*^MWT^* mice) were used as controls. Mice were randomly allocated to experimental groups. Mouse experiments were performed according to protocols approved by the animal ethics committee of Sir Run Run Shaw Hospital, Zhejiang University School of Medicine (No. SRRSH202102039).

### Colitis Models

The DSS‐induced colitis model was established as previously described.^[^ ^25^ ^]^ For butyrate administration, 150 mm sodium butyrate (Sigma–Aldrich, St. Louis, MO, USA) was supplemented in drinking water from day 0 of DSS (MP Biomedicals, Santa Ana, CA, USA) treatment.

For TNF‐α neutralization, mice were intraperitoneally (i.p.) injected with anti‐mouse TNF‐α antibody (150 µg per mouse, BioXcell, Lebanon, NH, USA) on day 1,3,5 after DSS administration.

### PHZ‐Induced Anemia

Mice were pretreated with 200 mm butyrate in drinking water for two days, followed by i.p. injection of 40 mg k^−1^g PHZ (Sigma–Aldrich, St. Louis, MO, USA) daily for two consecutive days.

### Sepsis Model

Mice were pretreated with 150 mm butyrate in drinking water for 2 days, followed by i.p. injection of LPS (5 mg k^−1^g) for 6 h.

### Low‐Iron Diet Model

Mice were fed a low‐iron diet containing 8 ppm iron (Jiangsu Xietong Pharmaceutical Bio‐engineering Co., Ltd, Nanjing, China) or a control diet containing 45 ppm iron (Jiangsu Xietong Pharmaceutical Bio‐engineering) after weaning for four weeks. During this phase, 100 mm sodium butyrate was supplemented in drinking water.

### Patient Specimens

Serum and colonic mucosa samples from IBD patients, and serum from healthy subjects were collected at SRRSH IBD Biobank in China (SRRSH‐IBC), Sir Run Run Shaw Hospital, Zhejiang University School of Medicine. For patients who received anti‐TNF‐α (infliximab) therapy, serum, and colonic mucosa samples were harvested before treatment initiation. The primary non‐response (PNR) to infliximab was assessed as previously described.^[^ ^26^ ^]^ Experiments involving patients’ specimens were performed under the approval of the Medical Ethics Committee of Sir Run Run Shaw Hospital, Zhejiang University School of Medicine (No.20210210‐24). Informed consent was obtained from all participants.

### Iron and Hemoglobin Measurement

Serum iron and colonic iron were measured using Iron Content Assay Kit (Solarbio, Beijing, China) according to the manufacturer's protocol. The intracellular iron in macrophages was evaluated using Iron Assay Kit (Sigma–Aldrich). Hemoglobin levels were measured on a BC‐6800 Auto Hematology Analyzer (Mindray, Shenzhen, China) following the manufacturer's instructions.

### Macrophage Depletion

To achieve macrophage depletion, mice were i.p. injected with 150 µL clodronate liposomes (FormuMax Scientific, Sunnyvale, CA, USA) on days −2, 2, and 5 of the DSS challenge.

### Murine Macrophage Isolation

Murine peritoneal macrophages and colonic macrophages were isolated and cultured as previously described.^[^ ^25^ ^]^ For the isolation of splenic macrophages and liver macrophages, spleens and livers of mice were cut into pieces and digested in DMEM/F‐12 medium containing 5% FBS and 300 U mL^−1^ collagenase IV at 37 °C for 30 min with gentle shaking. The digested tissues were passed through 70‐µm cell strainers to get a single‐cell suspension. Macrophages were magnetically sorted on an EasyEights EasySep Magnet (STEMCELL, Cambridge, MA, USA) using EasySep Mouse F4/80 Positive Selection Kit (STEMCELL). The purity of isolated macrophages (CD11b^+^F4/80^+^) was >98% (for peritoneal macrophages) or >92% (for colonic, liver, and splenic macrophages) as validated by flow cytometry. Macrophages were cultured in DMEM/F‐12 medium supplemented with 10% FBS, 100 U mL penicillin (Thermo Fisher Scientific, Waltham, MA, USA), and 100 µg mL streptomycin (Thermo Fisher Scientific).

### Human Macrophage Differentiation

PBMCs were isolated from the peripheral blood of healthy donors using Ficoll density gradient centrifugation (Solarbio, Beijing, China). The isolated PBMCs were cultured in a six‐well plate with RPMI 1640 medium containing 10% FBS, 300 U mL^−1^ penicillin (Thermo Fisher Scientific), 300 µg mL^−1^ streptomycin (Thermo Fisher Scientific), 10 mm HEPES buffer (Thermo Fisher Scientific) and 25 ng mL^−1^ human M‐CSF (Peprotech). On day 3, cells were replenished with fresh medium. On day 7, the adherent, mature macrophages were trypsinized and reseeded in 24‐well plates for butyrate treatment.

### ChIP

The ChIP assay was performed using SimpleChIP Enzymatic Chromatin IP Kit (Magnetic Beads) according to the manufacturer's protocols (#9003, Cell Signaling Technology). In brief, peritoneal macrophages were treated with PBS or 1 mm butyrate. Eight hours later, cells were harvested, and protein and DNA were cross‐linked with 1% formaldehyde for 15 min at room temperature before glycine neutralization, followed by DNA fragmentation by sonication. The protein‐DNA complexes were immunoprecipitated with anti‐acetyl‐Histone H3 (Lys18) antibody (#9675S, Cell Signaling Technology) or normal rabbit IgG (#2729, Cell Signaling Technology) at 4 °C overnight on a rotator. Chromatin elution and DNA purification were performed using buffers contained in the kit. The purified DNA samples were subjected to QPCR analysis. The primer sequences for *Slc40a1* promoter were: forward–5′‐CTAGCACCTGCCAAACGACT‐3′; reverse–5′‐GGCAGCGGCTTATAGGGAG‐3′.

### Cytokine Array

Peritoneal macrophages were pre‐treated with butyrate for 6 h, then stimulated with 1 µg mL^−1^ LPS for 12 h. Cell culture supernatants were centrifuged at 400 g for 5 min at 4 °C. The supernatants were subjected to cytokine analysis using Raybiotech Protein Array (Norcross, GA, USA).

### RNA Sequencing

Peritoneal macrophages were stimulated with 2 mm butyrate for 12 h before Trizol lysis. Cell lysates were sent to Biomarker Technologies (Beijing, China) and subjected to RNA sequencing. The data was submitted to the NCBI Sequence Read Archive under the accession number PRJNA974685.

### SCFA Quantification

Serum samples from IBD patients or healthy donors were used to determine SCFA production. Sample preparation and SCFA extraction were performed according to a previous study.^[^ ^27^ ^]^ The gas chromatography–mass spectrometry (GC‐MS) analysis was performed on a Shimadzu GCMS‐QP2010 Ultra equipped with a capillary column (Agilent Technologies, Santa Clara, CA, USA).

### Quantitative Polymerase Chain Reaction (QPCR)

Total RNA from colon tissues or in vitro‐cultured macrophages was extracted using the Trizol method, then was reversely transcribed to cDNA using ReverTra Ace Kit (Toyobo, Osaka, Japan). QPCR was performed using SYBR Green Reagent (CwBio, Beijing, China). Gene expression were analyzed using the 2^−ΔΔCT^ method and normalized to the mRNA levels of β‐actin. The primer sequences are listed in Table S2 (Supporting Information).

### Flow Cytometry

Single‐cell suspension from mouse colon tissues was prepared as previously described,^[^ ^24a^ ^]^ and then incubated with TruStain FcX PLUS (anti‐mouse CD16/32) Antibody (Biolegend, San Diego, CA, USA) on ice for 15 min. Thereafter, cells were incubated with anti‐Ferroportin antibody (Novus Biologicals, Centennial, CO, USA) at 4 °C for 20 min, washed twice with PBS, followed by staining with FITC Donkey anti‐rabbit IgG antibody (Biolegend), APC/Cyanine7 anti‐CD45 antibody (Biolegend), APC anti‐F4/80 Antibody (Biolegend), and Zombie Violet Viability dye (Biolegend) at 4 °C for 20 min in dark. After washing with PBS, cells were subjected to flow cytometry analysis on a NovoCyteTM Flow Cytometer (Agilent, Santa Clara, CA, USA).

### Enzyme‐Linked Immunosorbent Assay (ELISA)

The levels of murine or human TNF‐α were evaluated by murine TNF‐α Standard TMB ELISA Development Kit (Peprotech), or human TNF‐α Mini TMB ELISA Development Kit (Peprotech) respectively according to the manufacturer's protocol.

### Immunoblotting

Immunoblotting was performed as previously described.^[^ ^7^ ^]^ The following antibodies were used: anti‐acetyl‐histone H3 (Cell Signaling Technology, Danvers, MA, USA), anti‐histone H3 (Cell Signaling Technology), anti‐ferroportin (Novus Biologicals). anti‐β‐Actin (Cell Signaling Technology).

### Co‐Immunoprecipitation

Peritoneal macrophages were treated with 1 mm butyrate or PBS for 4 h. Cells were lysed using immunoprecipitation lysis buffer (Beyotime) on ice for 30 min, the supernatants were obtained by centrifugation. Protein A magnetic beads were incubated with primary anti‐Sp1 rabbit mAb (Cell Signaling Technology, 1:50) by rotating for 20 min at room temperature. After washing with PBS‐T, the antibody‐conjugated beads were incubated with cell lysates at 4 °C overnight. Then the beads were washed and eluted using immunoprecipitation lysis buffer. The products were immunoblotted with anti‐Sp1 mouse mAb (Santa Cruz Biotechnology, Dallas, TX, USA, 1:1000) and anti‐HDAC1 mouse mAb (Cell Signaling Technology, 1:1000).

### Calcein‐AM Staining

Calcein‐AM solution (Biolegend) was added to the macrophage culture medium at a final concentration of 20 nm. Macrophages were then incubated in the presence of calcein‐AM for 30 min at 37 °C, harvested by cell scrapers, and centrifuged at 400 g for 5 min at 4 °C. After resuspending in pre‐warmed DMEM/F12 medium, calcein‐AM labeled macrophages were analyzed for intracellular iron contents on a flow cytometer (BD FACSCalibur, Franklin Lakes, NJ, USA).

### Statistical Analysis

Data were analyzed using GraphPad Prism 8.0 Software (GraphPad Software, Inc.) and were presented as mean ± standard error of measurement (SEM). Unpaired, two‐tailed Student's *t*‐test was used to compare the differences between the two groups. Spearman's correlation test was used to analyze the correlation between two parameters. *p* < 0.05 was considered statistically significant.

### Ethics Approval Statement

Animal studies were conducted according to protocols approved by the Animal Ethics Committee of Sir Run Run Shaw Hospital, Zhejiang University School of Medicine (No. SRRSH202102039). Experiments involving human specimens were performed under the approval of the Medical Ethics Committee of Sir Run Run Shaw Hospital, Zhejiang University School of Medicine (No.20210210‐24).

## Conflict of Interest

The authors declare no conflict of interest.

## Author Contributions

P.X. and X.C. contributed equally to this work. P.X. and Q.C. were associated with concept study and design. X.C., Z.Z., K.G., M.S., Y.H., Y.Z., L.H., Y.Z., M.X., Y.Z., Z.H., P.X., X.X., J.L., and L.Y. performed experiments and data analysis. X.C., P.X., and Q.C. wrote the final manuscript. Q.C., R.L., L.Y., Z.Z., and Y.Z. performed clinical sample collection and processing. P.X., Q.C., R.L., M.X., Z.H., Y.K., and Z.S. provided financial or technical support.

## Supporting information

Supporting Information

## Data Availability

The data that support the findings of this study are available from the corresponding author upon reasonable request.
