# Supplementary material for: Butyrate Prevents the Pathogenic Anemia‐Inflammation Circuit by Facilitating Macrophage Iron Export
Source: Adv Sci (Weinh). 2024 Jan 18;11(12):2306571. doi: 10.1002/advs.202306571 (PMC10966513; doi:10.1002/advs.202306571)
Supplement: Supplementary file 1 — Supporting Information [file ADVS-11-2306571-s001.pdf]

ADVANCED SCIENCE
Open Access

Supporting Information

for *Adv. Sci.*, DOI 10.1002/advs.202306571

Butyrate Prevents the Pathogenic Anemia-Inflammation Circuit by Facilitating Macrophage Iron Export

Peng Xiao, Xuechun Cai, Zhou Zhang, Ke Guo, Yuehai Ke, Ziwei Hu, Zhangfa Song, Yuening Zhao, Lingya Yao, Manlu Shen, Jingyun Li, Youling Huang, Lingna Ye, Lingjie Huang, Yu Zhang, Rongbei Liu, Mengque Xu, Xutao Xu, Yuan Zhao and Qian Cao**

Supporting Information

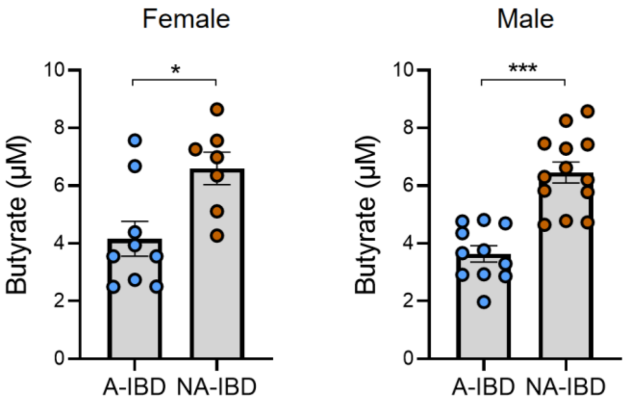

Figure S1. Serum butyrate concentrations were analyzed in male and female cohorts separately. Unpaired, two-tailed Student's *t* test, *$p < 0.05$; ***$p < 0.001$.

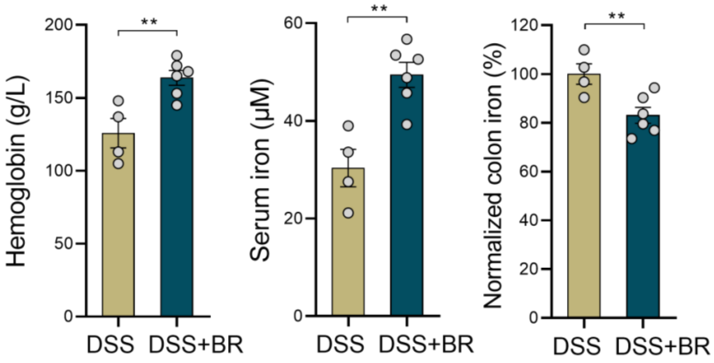

Figure S2. Butyrate improves anemia in nude mice. Athymic nude mice were challenged with 2.5% DSS with or without butyrate administration (n= 4 to 7/group). The levels of hemoglobin, serum iron, and colonic iron were evaluated. Unpaired, two-tailed Student's *t* test, **$p < 0.01$.

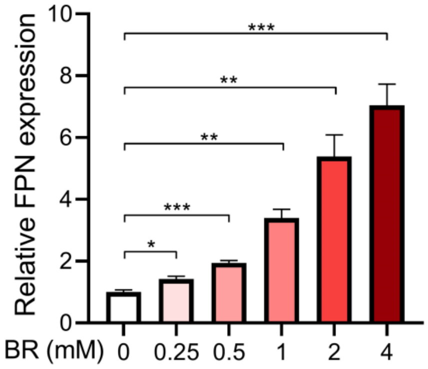

Figure S3. Peritoneal macrophages were treated with indicated concentrations of butyrate for 12 hours, the expression of FPN was evaluated by QPCR. *p < 0.05; **p < 0.01; ***p < 0.001, unpaired, two-tailed Student's *t* test.

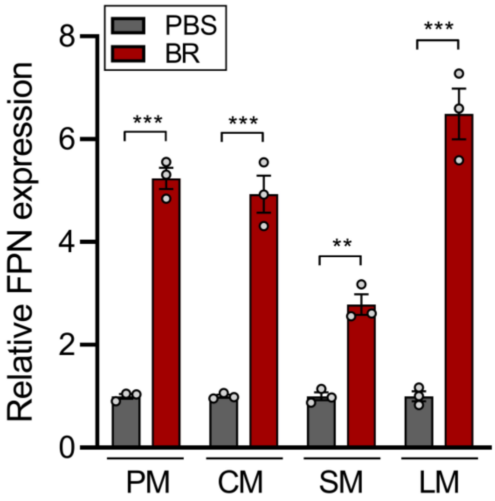

Figure S4. PM, colonic macrophages (C-Mac), splenic macrophages (S-Mac), and liver macrophages (L-Mac) from healthy mice were treated with 1 mM butyrate for 12 hours, the expression of FPN was evaluated by QPCR. **p < 0.01; ***p < 0.001, unpaired, two-tailed Student's *t* test.

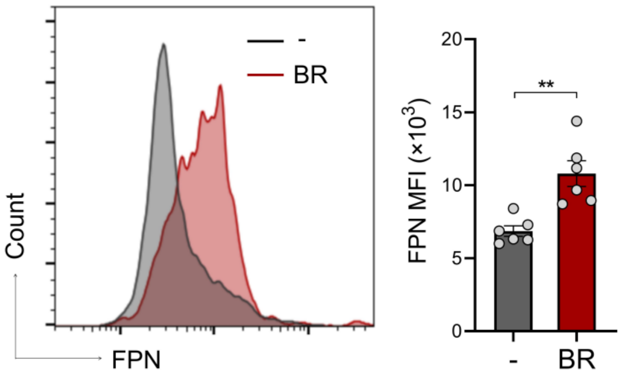

Figure S5. Mice were fed 2.5% DSS with or without 150 mM butyrate in drinking water for 8 days, the protein levels on CD45$^+$CD11b$^+$F4/80$^+$ colonic macrophages were evaluated by flow cytometry (n = 6/group). Left, representative plot. Right, statistical analysis. **p < 0.01; unpaired, two-tailed Student's *t* test.

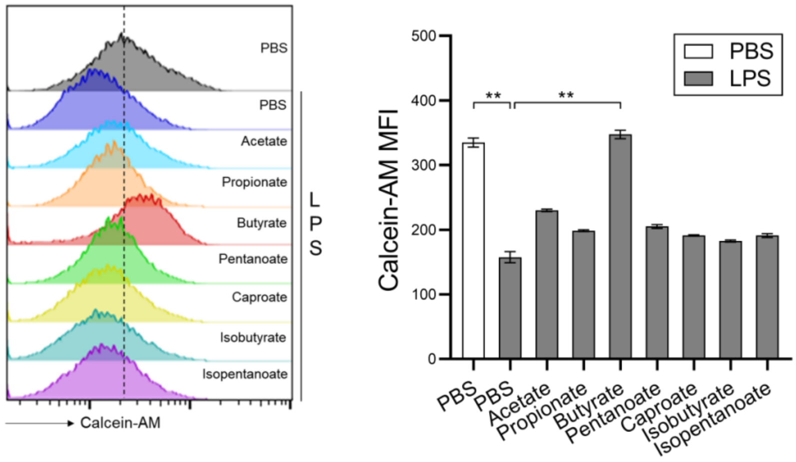

Figure S6. PMs were pre-treated with 1 mM indicated SCFAs for 10 hours, followed by 1 μg/ml LPS treatment. The fluorescence intensity of calcein-AM was evaluated by flow cytometry. Unpaired, two-tailed Student's *t* test, **p < 0.01.

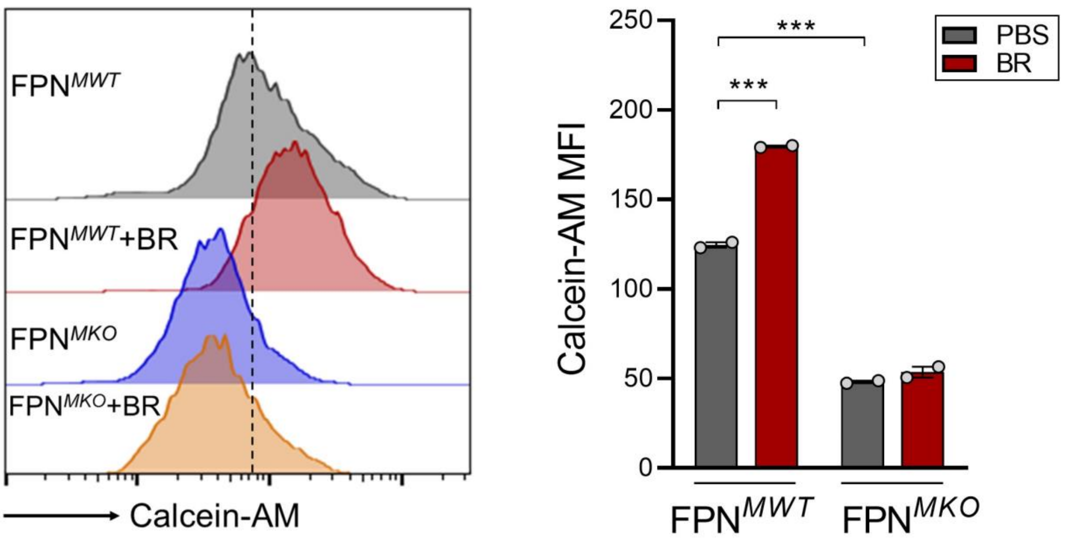

Figure S7. PMs from FPN^{MWT} and FPN^{MKO} mice were pretreated with 1 mM butyrate for 4 hours, followed by 1 μ g/ml LPS stimulation overnight. The fluorescence intensity of calcein-AM was evaluated by flow cytometry. Unpaired, two-tailed Student's *t* test, ***p < 0.001

Table S1

Patient characteristics. n.s = not significant; SD = Standard deviation.

	Healthy (n = 10)	A-IBD (n = 20)	NA-IBD (n = 20)	P-value (A-IBDvsNA-IBD)
Subtype (CD/UC)	N/A	10/10	10/10	/
Sex (Female/Male)	3/7	9/11	7/13	/
BMI (mean ± SD)	N/A	19.3±2.6	20.9±2.9	>0.5, n.s
Age (mean ± SD)	35.3±7.7	32.6±12.3	36.3±12.5	>0.5, n.s
Smokers/Non-smokers	N/A	2/18	4/16	/
Family history (Yes/No)	/	0/20	0/20	/
Disease Duration (mean year± SD)	/	3.8±3.9	4.8±4.7	>0.5, n.s
Perianal involvement [CD] (Yes/No)	/	9/1	9/1	/
Extraintestinal manifestations (Yes/No)	/	9/11	12/8	/
**Montreal classification**				
A1/A2/A3 [CD]	/	1/7/2	0/8/3	/
L1/L2/L3/L4 [CD]	/	0/1/8/1	1/2/6/1	/
B1/B2/B3 [CD]	/	8/2/0	8/0/2	/
E1/E2/E3 [UC]	/	1/2/7/	2/4/4/	/

Table S2

Primer Sequences (5’-3’)

mouse β-Actin F	GGCTGTATTCCCCTCCATCG
mouse β-Actin R	CCAGTTGGTAACAATGCCATGT
mouse FPN F	TTGCAGGAGTCATTGCTGCTA
mouse FPN R	TGGAGTTCTGCACACCATTGAT
human β-Actin F	CATGTACGTTGCTATCCAGGC
human β-Actin R	CTCCTTAATGTCACGCACGAT
human FPN F	CTACTTGGGGAGATCGGATGT
human FPN R	CTGGGCCACTTTAAGTCTAGC
